# A Comparative Systematic Literature Review on Knee Bone Reports from MRI, X-Rays and CT Scans Using Deep Learning and Machine Learning Methodologies

**DOI:** 10.3390/diagnostics10080518

**Published:** 2020-07-26

**Authors:** Hafsa Khalid, Muzammil Hussain, Mohammed A. Al Ghamdi, Tayyaba Khalid, Khadija Khalid, Muhammad Adnan Khan, Kalsoom Fatima, Khalid Masood, Sultan H. Almotiri, Muhammad Shoaib Farooq, Aqsa Ahmed

**Affiliations:** 1 Department of Computer Science, School of Systems and Technology, University of Management and Technology, Lahore 54000, Pakistan; muzammil.hussain@umt.edu.pk (M.H.); F2018108011@umt.edu.com (K.F.); shoaib.farooq@umt.edu.pk (M.S.F.); 2 Department of Computer Science, Umm Al-Qura University, Makkah 715, Saudi Arabia; maeghamdi@uqu.edu.sa (M.A.A.G.); shmotiri@uqu.edu.sa (S.H.A.); 3 Gynecology Department of Shahdra Hospital, Lahore 54000, Pakistan; doctayyaba.khalid@yahoo.com; 4 Department of Electrical Engineering, School of Engineering, University of Management and Technology, Lahore 54000, Pakistan; khadijakhalid.ee@gmail.com; 5 Department of Computer Science, Lahore Garrison University, Lahore 54000, Pakistan; madnankhan@lgu.edu.pk (M.A.K.); khalid.masood@lgu.edu.pk (K.M.); 6 Department of Botany, University of Agriculture, Faisalabad 38000, Pakistan; Ahmedaqsa686@gmail.com

**Keywords:** magnetic resonance imaging (MRI), computed tomography (CT scan), electromagnetic radiation (X-ray), trabecular bone (TB)

## Abstract

The purpose of this research was to provide a “systematic literature review” of knee bone reports that are obtained by MRI, CT scans, and X-rays by using deep learning and machine learning techniques by comparing different approaches—to perform a comprehensive study on the deep learning and machine learning methodologies to diagnose knee bone diseases by detecting symptoms from X-ray, CT scan, and MRI images. This study will help those researchers who want to conduct research in the knee bone field. A comparative systematic literature review was conducted for the accomplishment of our work. A total of 32 papers were reviewed in this research. Six papers consist of X-rays of knee bone with deep learning methodologies, five papers cover the MRI of knee bone using deep learning approaches, and another five papers cover CT scans of knee bone with deep learning techniques. Another 16 papers cover the machine learning techniques for evaluating CT scans, X-rays, and MRIs of knee bone. This research compares the deep learning methodologies for CT scan, MRI, and X-ray reports on knee bone, comparing the accuracy of each technique, which can be used for future development. In the future, this research will be enhanced by comparing X-ray, CT-scan, and MRI reports of knee bone with information retrieval and big data techniques. The results show that deep learning techniques are best for X-ray, MRI, and CT scan images of the knee bone to diagnose diseases.

## 1. Introduction 

This section gives a brief introduction to our study to elaborate on the all-important aspects related to the knee bone, knee bone diseases, and screening techniques, i.e., MRI, CT scans, and X-rays. This section also explains the importance of deep learning and machine learning techniques for medical image processing. The following subsections constitute an overview of our research.

### 1.1. Structure of Human Knee

There are many joints in the human body, but the knee joint has a very important role among all the joints as it is the largest joint. Its purpose is to provide a crucial point between the thigh and the lower leg during development. It comprises bones (the femur, tibia, and patella), which are connected by the articular surfaces of the hyaline ligament (tibiofemoral and patellofemoral).

**Figure 1:**
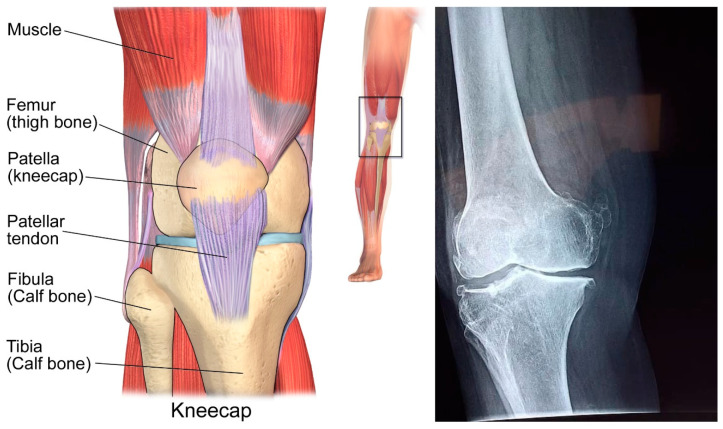
Structure of human knee.

The femur cooperates with the tibia in two important zones: average (closer to the midline of the body) and parallel (far from the middle). Figure 1 demonstrates an attractive reverberation picture of the knee in a sagittal plane from an anatomical chartbook for reference. The ligament is noticeable as a splendid thin layer covering the bones.

The fat in this picture has been overemphasised, to make the ligament more conspicuous among the encompassing tissues. Splendid mass adjoins the femoral and tibial ligaments in the muscles [1].

### 1.2. Osteoarthritis

Osteoarthritis is the most widely recognized type of joint pain in the knee. It is a degenerative, “wear-and-tear” sort of joint pain that happens regularly in individuals that are 50 years old and older; however, it may happen in more youthful individuals as well. In osteoarthritis, the ligament in the knee joint erodes step by step.

It is believed that as many as 30% of the general population older than 65 will eventually develop Osteoarthritis OA over time [1].

### 1.3. Screening Techniques

Tragically, the standard treatment for OA today does not completely fix the malady. It is subsequently of most significance to detect the degeneration of the ligament at the beginning before it becomes irreversible.

There are several approaches to determining the level of ligament degeneration in patients.

#### 1.3.1. Radiography (X-ray)

Over the previous decades, X-rays of the joint space width (JSW) have been the customary technique for OA screening. They offer significant advantages over arthroscopy since they are non-obtrusive and can be performed again if necessary. Among the inconveniences of X-ray imaging is the lack of accuracy for momentary examinations, because of the way in which changes in the ligament must be determined through X-ray pictures over 2–3 years [1].

#### 1.3.2. Magnetic Resonance Imaging (MRI)

MRI is an almost new standard strategy for the screening of the ligament since it does not utilize ionizing radiation, is non-intrusive and repeatable, and gives decent picture quality with high contrast and detail. X-rays convey pictures in an advanced format, which can be stored and effectively recovered, and offer an assortment of parameters for ideal picture procurement. The drawbacks include the staggering expense of the device (particularly for high field quality magnets), the long examination times, and the inclination to image ancient rarities.

#### 1.3.3. Computed Tomography (CT scan)

A CT scan is just like an X-ray that produces cross-sectional pictures of a particular site in one’s body. For instance, a CT output of one’s knee would enable specialists to analyze illness or investigate wounds on one’s knee.

A CT scanner circles the body and sends pictures to a PC. The PC utilizes these pictures to make the point by point pictures. This enables specialists and preparedness experts to see the muscles, ligaments, tendons, vessels, and bones that make up one’s knee.

A CT scan is, likewise, sometimes alluded to as a CAT scan. The output is obtained at a clinic or a specific outpatient testing office.

### 1.4. Deep Learning in Medical Image Processing

In previous years, deep learning gained immense consideration by showing promising outcomes for some best-in-class methodologies, for example, discourse acknowledgment, manually written character acknowledgment, picture characterization, identification, and division. There are desires to use deep learning to enhance or create restorative picture examination applications, for example, PC-assisted conclusions, picture enlistment, multi-modular picture investigation, picture division, and recovery. There have been some applications that utilize deep learning in restorative applications like cell following and organ disease location. Specialists utilize attractive reverberation pictures as successful instruments to determine infections [2].

### 1.5. Machine Learning in Medical Image Processing

Machine learning is an incredible method for identifying patterns in therapeutic pictures; be that as it may, it must be utilized with caution since it may very well be abused if the qualities and shortcomings of this innovation are not comprehended.

Machine learning is a procedure for identifying patterns that can be associated with medical pictures. Although it is an amazing asset that can help in drawing medical conclusions, it tends to be biased. Machine adaptation regularly starts with the machine learning calculation framework determining the picture inclusions that are accepted to be of significance in making the forecast or coming to a conclusion. The machine learning calculation framework at that point recognizes the best mix of these picture highlights for ordering the picture or determining some measurement for the given picture locale. There are a few techniques that can be utilized, each with various qualities and shortcomings. There are open-source forms of the greater part of these machine learning strategies that make them simple to attempt and apply to pictures. A few measurements for estimating the execution of a calculation exist; in any case, one must know about the conceivably related entanglements that can bring about deluding measurements. Recently, machine learning has begun to be utilized more often; this strategy has the advantage that it does not require picture highlight distinguishing and computation as an initial step; rather, highlights are recognized as a component of the learning procedure. Machine learning has been utilized in restorative imaging and will have a more prominent impact in the future. Those working in medical imaging must know about how machine learning functions [3].

### 1.6. Purpose of the Study

Machine and deep learning algorithms are quickly developing a unique exploration of clinical imaging. These techniques have a huge amount of algorithms that can be used for medical imaging to detect the symptoms of diseases at very early stages. These programming techniques use supervised and unsupervised algorithms that can predict diseases from medical images—i.e., X-rays MRIs, and CT scans—by using a huge number of datasets. To date, considerable endeavors have been made for the improvement of clinical imaging applications utilizing these algorithms to analyze the mistakes in infection indicative frameworks that may bring about very vague clinical indications. This paper gives a review and comparison of clinical imaging in the machine and deep learning strategies to unambiguously dissect diseases of the knee bone. It conveys thoughts concerning the set-up of these algorithms that can be utilized for the examination of diseases and programmed decision making. Some techniques can provide the best results by using machine learning methodologies, and some can provide better accuracy by using deep learning methodologies. Thus, in this review paper, we compare the deep and machine learning algorithms with different forms of medical imaging—i.e., X-rays, CT scans, and MRI—to conclude which techniques are more accurate for this imaging.

### 1.7. Reviews and Hypothesis

We reviewed 32 papers for our comparison. From these, six papers relate to deep learning techniques for knee bone X-rays, five papers relate to deep learning techniques for MRI, and five relate to CT scans with deep learning techniques. Similarly, we selected six papers for X-ray imaging with machine learning techniques, five for CT scan imaging, and five for MRI with machine learning techniques.

We extracted image types, datasets, knee bone disease statuses, and accuracies from each paper regarding deep learning and machine learning. For the results, we compared the accuracy of these techniques and determined the results by calculating their average percentages. According to our results, we concluded that deep learning techniques provide more accuracy than machine learning for X-ray, MRI, and CT scan images of knee bones.

## 2. Related Work

A lot of work has performed in the knee bone field by using reported data of MRI, X-rays, and CT scans by using the deep learning and machine learning techniques, some of which is described below.

In the present review, the authors describe methodology for naturally diagnosing and reviewing knee OA from plain radiographs. Rather than past examinations, their model uses explicit highlights significant for the ailment. Besides, considering the recently described methodologies, their technique accomplishes the best multi-class grouping results, despite having an alternate testing set that shows 66.7% precision [4].

The researchers of this research created and assessed a programmed 3D deformable methodology for knee MRI having force inhomogeneity. They showed that the underlying point can be resolved, depending on the histogram from earlier learning. It is additionally striking that no preparation stage is required, dissimilarly to other custom deformable models—for example, Active Shape Model (ASM), Active Appearance Model (AAM), and map book-based models. The exploratory outcomes showed that their methodology accomplishes a 95% Dice, 93% social epistemic network signature (SENS), and 99% Standard Performance Evaluation Corporation (SPEC) in volume assessment but an Average symmetric surface distance (ASSD) of 1.17 mm and Root *mean* square symmetric surface distance (RMSD) of 2.01 mm in surface assessment [5].

This work shows the credibility of the program’s bone division and characterization of CT scans utilizing a convolutional neural network (CNN). The model accomplishes a high Diverse Counterfactual Explanations (Dice) coefficient and ends up being very robust to Gaussian clamor. In any case, a few constraints and enhancements that would unquestionably improve the execution are distinguished. An enhanced yet comparative model could be helpful in a few clinical and research applications for numerous therapeutic undertakings [6].

Researchers study the programmed characterization of complete tissues with 3D MRI. With an MRI flag structure, there is no need for unfolding. The additional data separated from the stage give better division than just utilizing greatness highlights. A request of greatness increment is acquired by diminishing the number of pixels that should be characterized [7].

Much more work has done in the field of deep and machine learning for the past decade, and in the future, there is a chance that deep learning, machine learning, big data, and information retrieval will remain the most tempting areas for researchers in the medical and engineering fields [8,9,10,11,12,13,14,15].

## 3. Systematic Literature Review 

A comparative “Systematic Literature Review” (SLR) has been chosen as the exploration technique. This paper utilizes SLR rules, which are a type of auxiliary investigation that utilizes a very much characterized technique. The SLR strategy is intended to be as reasonable as conceivable by being adaptable and repeatable. As per [16], the purpose of an SLR is to give a complete-as-possible overview of all studies that are identified within a certain branch of knowledge. In the meantime, customary surveys endeavor to condense the implications of various studies.

**Figure 2:**
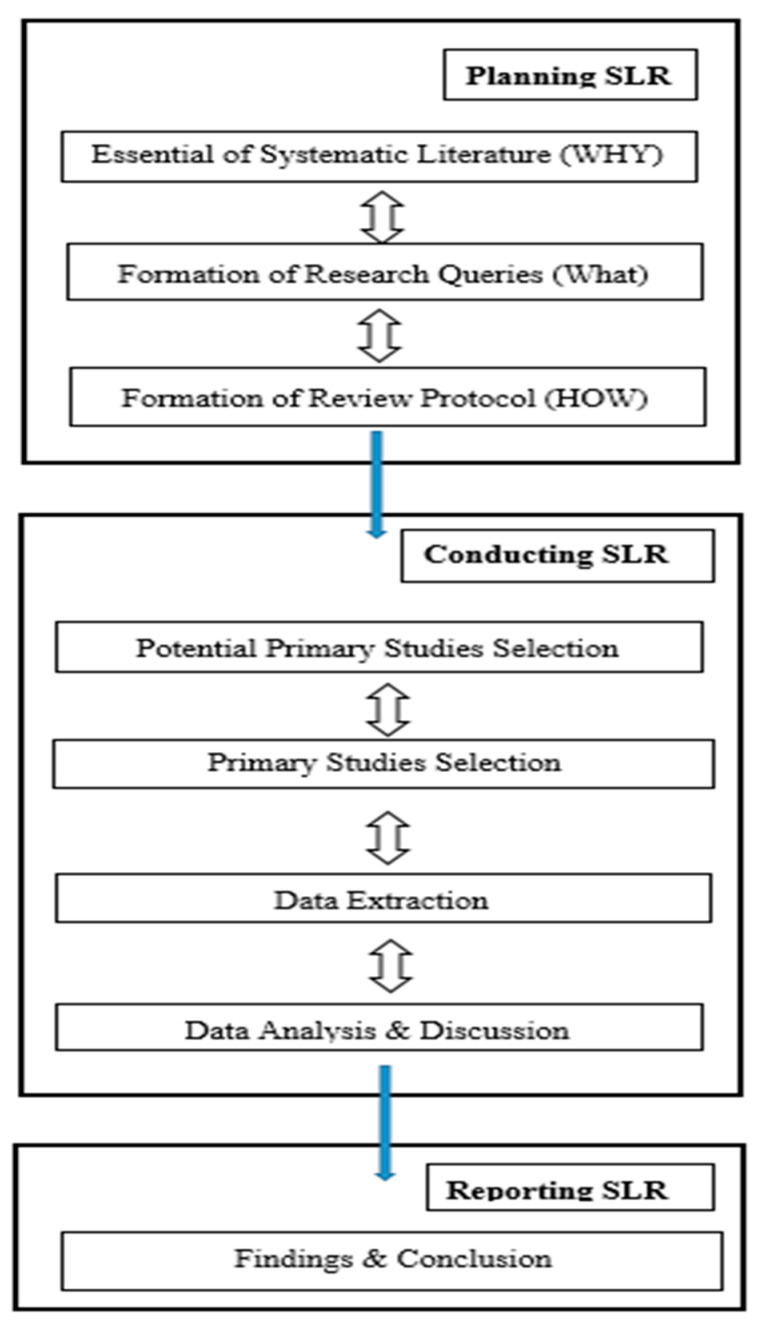
The systematic literature review process.

We performed an SLR utilizing the rules given in [17]. The SLR is an examination strategy for performing a survey efficiently capturing all characterized advances [17]. There are generally three stages of an SLR: planning the SLR, directing the SLR, and reporting the SLR (see Figure 2). Below, we discuss how we performed each part in these three stages.

According to [16], an SLR procedure is secured by three back-to-back stages: planning the SLR, conducting the SLR, and reporting the SLR. In this section, we will concentrate on the arranging stage, which includes characterizing the study goals and how the audit was done.

### 3.1. Planning SLR

The following paragraph describes the complete plan for our systematic literature review.

#### 3.1.1. Necessity of the SLR

The purpose of Evidence-Based Software Engineering (EBSE) is to aggregate the best results from research and examine these with regard to (reality) observations to assess these issues [17].

We utilized the following inquiry string to prove that there existed no comparative study in the literature.

((‘Knee bone’ OR ‘Deep Learning’ OR ‘Machine Learning’) AND (‘Knee bone’ OR MRI’) AND (‘Knee bone’ OR CT Scan’) AND (‘Knee bone’ OR X-ray’) AND (‘MRI’ OR ‘Deep Learning’) AND (‘X-ray’ OR ‘Deep Learning’) AND (‘CT Scan’ OR ‘Deep Learning’) AND (‘MRI’ OR ‘Machine Learning’) AND (‘X-ray’ OR ‘Machine Learning’) AND (‘CT Scan’ OR ‘Machine Learning’) AND (‘Systematic Review’ OR ‘Comparative Study Review’).

The recognized examinations were investigated based on their titles, concepts, and ends. The outcome demonstrated that there was no other SLR that had a similar degree and time frame of distribution.

#### 3.1.2. Research Questions

We characterized our research questions (RQ) as shown in Table 1 below.

#### 3.1.3. Review Protocol

This section introduces the survey convention that we characterized for directing the SLR. Below, we describe how we planned the SLR, choice of studies, information extraction, and information examination.

##### Search Process

It is very difficult to form an effective search string for primary studies. That is why we firstly focus on our main domain of knee bone images with deep learning and machine learning techniques and then focus on the image type, i.e., MRI, CT scan, or X-ray.

Then, we consider hyphens, antonyms, and synonyms for each word. Finally, we used Boolean operators (‘AND’ and ‘OR’) and wildcards (‘*’) to form an authentic string for the search process.

Table 2 shows the combinations of keywords, Boolean operators, and wildcards. The population column shows the keywords with the ‘AND’ operator, and the intervention column shows the combinations of more keywords with the ‘OR’ operator.

“*“ shows that the alphabets would be the same till asterisk *, but after that any alphabet can occur for searching query.

##### Study Exclusion Criteria

The accessibility of the full content of the essential investigation and English language were imperative for choice. Short papers were excluded.

##### Study Inclusion Criteria

The basic criteria for paper selection were related to the research questions mentioned in Table 3. We settled on 32 quality papers to study, which met our requirements. The selected criteria were set to study all aspects of our research. The included papers and criteria are mentioned in Table 3 below.

##### Quality Criteria for Primary Studies

When leading an SLR, it is essential to choose investigations of high quality in determining solid outcomes and ends *. This requires great SLR management, the adoption of the right catchphrases, and an all-around characterized consideration of avoidance criteria. We connected the accompanying criteria for further breaking down the nature of concerns regarding their legitimacy (see Table 4).

##### Data Extraction

After studying the primary studies, we extracted the data into tables. The outcomes were recorded in the structures for further examination. Below, we give the meanings of explicit data we obtained in connection to RQs (see Table 5).

## 4. SLR Conduct 

After planning the study, we conducted our SLR. The following paragraphs describe the comprehensive details of our SLR methodology.

### 4.1. Design

In this section, we describe the design of the review by forming the strategies and queries to judge the quality of the measurements of this SLR.

#### 4.1.1. Research Queries

Describing and depicting profound learning with machine learning methods is another issue, since we found that the most recent related work was in 2019. Consistently, a couple of examinations have driven machine learning methods with profound learning in the knee bone medicinal field. From now on, this paper is intended to describe the features that impact the sufficiency of Deep Learning methodologies with machine learning for knee bone information from MRI, CT scans, and X-rasy. The SLR Research Question (RQ) that we intend to answer in this paper is as follows:

**“Which techniques of deep learning and machine learning are best for diagnosing knee bone diseases by using the imaging report data for MRI, CT scans, and X-rays?”.**

#### 4.1.2. Search Procedure

This SLR focuses on seeking logical databases as opposed to explicit books or specialized reports. A presumption was made that the majority of the exploration results in books and reports were likewise regularly depicted or referenced in logical papers. This study chose five databases to perform the SLR search process:

- www.scholar.google.com;
- www.sciencedirect.com;
- www.ieeexplore.ieee.org;
- www.springer.com;
- www.ieeexplore.ieee.org.

These databases were picked as they offer the most vital and most astounding full-content diaries and meeting procedures; 32 research papers were reviewed to cover the diagnosis of disease in knee bones by using deep learning and machine learning techniques.

The following keywords were used to find related studies to accomplish this SLR research:

**“Deep learning in knee bone” OR “Machine learning in knee bone” OR “Knee bone diseases “OR “Knee bone X-ray” OR “Knee bone MRI” OR “Knee bone CT-scan”.**

### 4.2. Study Selection

The determination of studies was performed through the following procedures [18]:

Inquiry across databases to identify significant studies, utilizing the search tags.

1. Discarding studies based on the avoidance criteria.
2. Discarding insignificantly concentrated studies based on the investigation of their titles and edited compositions.
3. Assessing the choice to concentrate, depending on a full content read.
4. Assessment by an outer specialist.
5. Re-examining the outcomes in irregular studies.
6. Acquiring essential examinations.

### 4.3. Quality Evaluation

As indicated by the rules of the SLR [18], three Quality Assurance (QA) queries must evaluate the nature of the examination of every proposition and provide a quantitative correlation between them. After selecting the primary studies according to the inclusion and exclusion criteria, we assessed their quality. The purpose of determining the quality of the papers was to determine how much our selected studies were relevant to our research questions and paper scope. We selected three quality questions and set their scores. We gave a score to each selected study, according to the relevancy of the paper to our scope. The scoring criteria are given below:

- Agree (A) = 1
- Some (S) = 0.5
- Disagree (D) = 0

The questions relating to the selected papers according to our requirements are given below:

- Do the selected papers refer to the required query?
- How are the function impediments archived?
- Were the detected research papers acceptable?

When the essential investigations of the SLR had been identified, we assessed them as indicated by the above queries. The score doled out to each examination for each inquiry is shown in Table 6 and Table 7 for deep learning and machine learning papers, respectively.

After obtaining the scores for these questions for all the selected studies, we determined the percentages by multiplying the averages of these question scores by 100. Let us take the example of SP1 for all three questions:

- Do the selected papers refer to the required query?

Our SP1 paper “Automatic knee osteoarthritis diagnosis from plain radiographs: a deep learning-based approach” is very relevant to our scope and required query. Because this paper is about the X-ray imaging of knee bone osteoarthritis with deep learning methodologies, we gave this paper a score of “1” because it fully relates to Question 1.

- How are the function impediments archived?

Although SP1 is quite good for our research, it is not supporting Question 2, so we gave it “0” for this question.

- Was the detected research paper acceptable?

Yes, this paper has authentic and quality research that is good enough for our research paper. Thus, we gave this paper a score of “1” for this question.

After getting the score, we determined the question score average for SP1 and then we multiplied it by 100 to get the percentage, i.e., 1 + 0 + 1 = 2 / 3 x 100 = 66.67%. In the end, we took the average of all the percentages according to % of an S1 + % of S2 +…………… % of S16 / 16 and then obtained the total percentage by multiplying the result by 100. Thus, we obtained the quality assessment percentages of the deep learning papers and machine learning papers.

Table 6 shows that our selected papers on deep learning techniques are highly related to our research requirements, as the relatedness scoring percentage is 70.83%.

Similarly, Table 7 indicates that our selected papers on machine learning techniques are highly related to our research requirements, as the relatedness scoring percentage is 76%.

Thus, it is obvious from the above two tables that our selected papers fulfill the quality requirements of the literature review.

### 4.4. Synthesis

Our research paper is related to knee bone reports, which we obtained by X-Ray, MRI, and CT scan imaging. We had to compare the data in these reports with the machine learning and deep learning methodologies. It was a challenge for us to find the studies that fitted our selection criteria because we required studies of deep learning methodologies with X-rays, MRI, and CT-scans and similarly required machine learning papers with these screening techniques. We found a lot of papers related to machine learning for X-rays, machine learning for knee bones, deep learning for CT scans, MRI images of knee bones, etc. However, it was very difficult to find papers that fulfilled our search queries, which are mentioned in Table 2.

Therefore, we decided to conduct our search on multiple levels, which are given below.

Level 1. Firstly, we selected databases to find selected research articles. There were almost 7574 related articles in different databases.

Level 2. At Level 2, we searched our papers in five databases including Springer, IEEE Xplore, Google Scholar, ACM, and Science Direct.

Level 3. At this level, we distinguished our papers related to deep learning and machine learning separately to perform a comparison between them.

Level 4. At Level 4, we took account of more papers related to MRI-, CT scan-, and X-ray-related deep learning and machine techniques.

Level 5. Finally, we found 32 papers, 16 on deep learning and 16 on machine learning. We distributed our papers in such a way that from these 30 papers on deep and machine learning, 10 papers were related to MRI, 12 consisted of X-rays, and the remaining 10 consisted of CT scans of knee bones.

**Figure 3:**
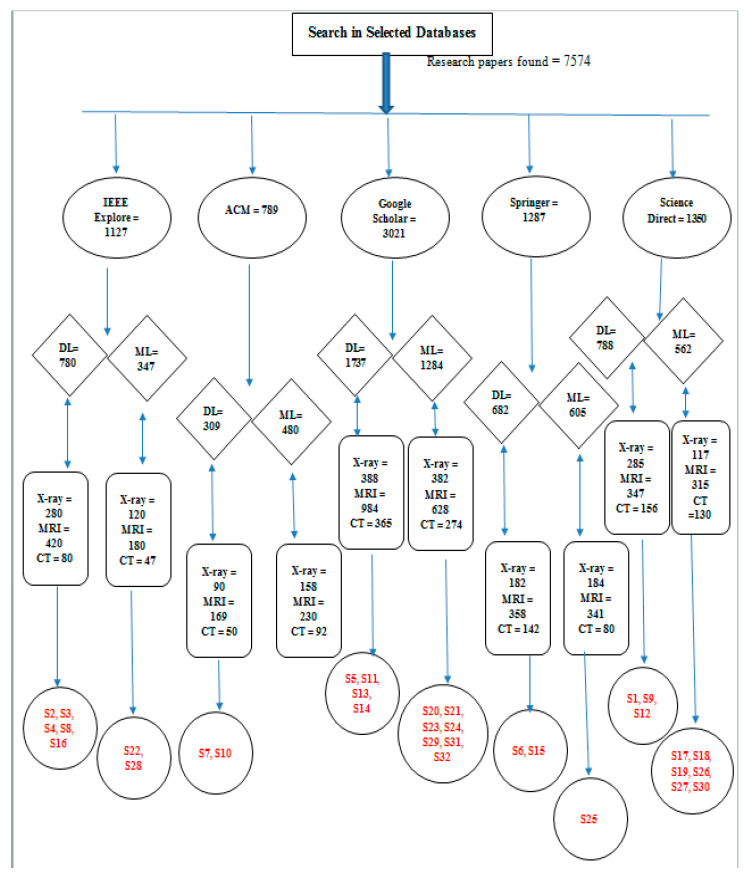
Hierarchy of number of Studies for SLR.

Figure 3 demonstrates the hierarchy of our research methodology, which consists of five levels:

### 4.5. Citation of Selected Papers

Table 8 shows the number of citations of selected research papers, which are taken from Google Scholar. Table 8 clearly shows that many papers are well-cited, which means that authentic papers were reviewed for this comparative literature review.

### 4.6. Years of Publication

**Figure 4:**
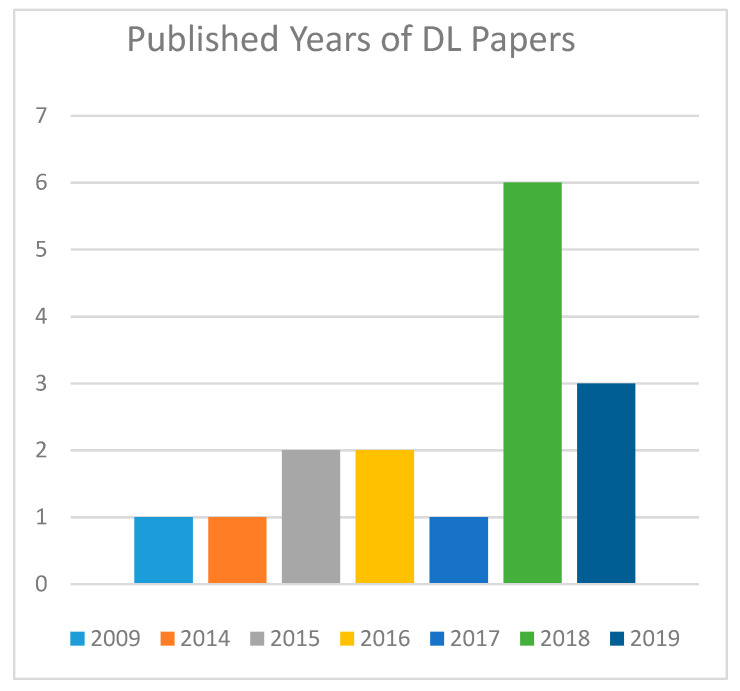
Publication by years of Deep Learning papers.

Figure 4 shows the numbers of essential examinations by year of distribution. These 16 articles are distributed across the years 2009, 2014, 2015, 2016, 2017, 2018, and 2019. It demonstrates that the year 2018 has more chosen articles than the other years.

According to Figure 4, the quantity of production was significantly in the year 2018 of studies utilizing Deep Learning approaches.

**Figure 5:**
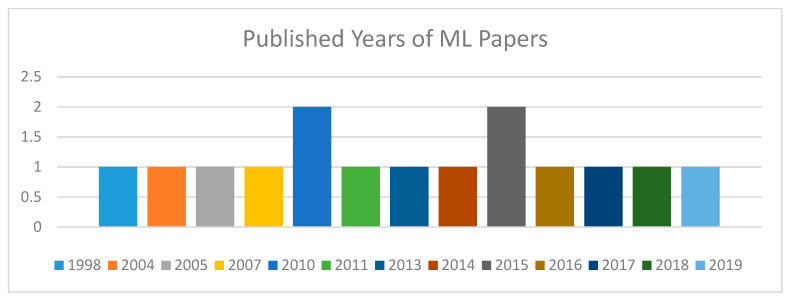
Published years of selected ML papers.

Figure 5 shows the published years of the selected machine learning papers. These papers are dispersed from the year 1998 to 2019. As papers related to our requirements of machine learning techniques for knee bone were rare in the previous 5 to 10 years, we decided to review more related papers; although they are so old, they were related to our requirements for this literature review. However, our major selected papers are from the last 10 years.

### 4.7. Data Extraction

Table 9 and Table 10 provide the information extraction frame that is utilized for all the chosen essential investigations to do a top-to-bottom study. It describes the author’s name of the paper, published year, image type, model, data set, disease, and accuracy of the proposed techniques.

Table 11 and Table 12 describe the work of the researchers that they have performed by using different methodologies in their research papers, predictions of their future work, and the advantages of their research.

Table 13 and Table 14 show the gaps in the review articles on deep learning and machine learning papers.

## 5. Discussion

Clinical imaging has prompted improvements in the determination and treatment of various ailments in children and adults. There are numerous sorts—or modalities—of clinical imaging systems, every one of which utilizes various advances and methods. Processed tomography (CT) and radiography (“regular X-beam” including mammography) both utilize ionizing radiation to produce pictures of the body. Ionizing radiation is a type of radiation that has enough power to possibly harm DNA and may raise an individual’s lifetime danger of malignant growths.

In CT, numerous X-ray images are recorded as the locator moves around the patient’s body. A computer recreates all the individual images into cross-sectional images or “cuts” of inner organs and tissues. A CT test includes a higher radiation portion than traditional radiography because the CT image is remade from numerous individual X-ray projections.

An MRI scanner can be utilized to take pictures of any piece of the body (e.g., the head, joints, midsection, legs, etc.), in any image bearing. X-rasy give better delicate tissue differentiation than CT and can better separate fat, water, muscle, and other delicate tissues than CT (CT is typically better for imaging bones). These pictures give data to doctors and can help in the diagnosis of a wide assortment of ailments and conditions.

A knee X-ray can help to elucidate the reasons for normal signs and side effects, for example, pain, sensitivity, swelling, or disfigurement of the knee. It can identify broken bones or a separated joint. After a damaged bone has been set, the picture can help to decide if the bone is in an inappropriate arrangement and whether it has recuperated appropriately.

Magnetic resonance imaging (MRI) of the knee utilizes an amazing attractive field, radio waves, and a computer to create point-by-point images of the structures inside the knee joint. It is commonly used to help analyze or assess pain shortcomings, growths, or seeping in and around the joint.

A computed tomography (CT) filter is a kind of X-ray approach that shows cross-sectional pictures of a particular zone on one’s body. For instance, a CT output of one’s knee would assist specialists with diagnosing maladies or reviewing wounds on one’s knee. A CT scanner circles the body and sends images to a computer.

A lot work has been performed in the field of knee bones on the basis of medical imaging.

The exact division of the articular ligaments from magnetic resonance (MR) pictures of the knee is significant for clinical examinations and medication preliminary investigations into conditions such as osteoarthritis. As of now, divisions are acquired utilizing tedious manual or self-loader calculations that have high between- and intra-eyewitness change abilities. This paper presents a significant advance towards performed programmed and precise divisions of the ligaments, a specific way of dealing with consequently sectioning the bones and concentrating on the bone–ligament interfaces (BCI) in the knee. The division is performed utilizing three-dimensional dynamic shape models, which are introduced utilizing a relative enlistment to a map book. The BCI are then extricated utilizing picture data and earlier information about the probability of each point having a place with the interface. The exactness and robustness of the methodology were tentatively proved utilizing an MR database of fat stifled ruined inclination review pictures. The femur, tibia, and patella bone divisions had middle Dice closeness coefficients of 0.96, 0.96, and 0.89, respectively, and a normal point-to-surface error of 0.16 mm on the BCI. The removed BCI had a middle surface cover of 0.94 with the genuine interface, showing its usefulness for ligament division or quantitative investigation [47].

In an earlier investigation of marathon runners, we saw that MR sweeps of the knee often indicated the hyperplasia of red (i.e., hematopoietic) bone marrow. Since the recurrence of this finding in different populaces is obscure, the reason for this investigation was to decide the overall prevalence of hematopoietic bone marrow hyperplasia in MR assessments of the knees of solid volunteers (n = 74), patients with side effects of knee issues (n = 54), and asymptomatic long distance runners (n = 23). The frequency of hematopoietic bone marrow hyperplasia was 3% (2/74) for the healthy volunteers, 15% (8/54) for the patients, and 43% (10/23) for the long distance runners. The distinction in the predominance between every one of the three gatherings was statistically significant at *p* < 0.05 for each situation with hematopoietic bone marrow hyperplasia; the distal femur was the main site influenced, while the epiphysis and proximal tibia were uninvolved. This example of influenced bone marrow with hyperplasia of the hematopoietic marrow might be valuable for differential analysis. We hypothesize that the high predominance of hematopoietic bone marrow hyperplasia in long distance runners may be a reaction to “sports frailty”, which is generally found in exceptionally adapted, vigorously prepared competitors [48].

Based on the above discussion of the benefits of medical imaging, we decided to select these imaging techniques—X-rays, MRI, and CT scans—for our research. Our first question in Section 3.1.2 was taken to cover this imaging for knee bones. The work on these image types in this paper is mentioned in Section 5.1.

This section discusses this SLR. The dialog is about the examination question referenced above in Section 3.1.2. The answers to the question are mentioned in the given paragraph.

### 5.1. Type of Knee Bone Images Used in SLR

Our first question was related to medical images of knee bone.

“Which sort of knee bone images were used for medical image processing in this literature review?”.

We asked this question to understand the focus of knee bone images by using different methodologies. We select three types of medical images for this SLR, which are X-rays, CT scans, and MRI images of knee bone. We studied six papers on knee bone X-rays with deep learning techniques and six papers on those with machine learning techniques. Then, we read five papers on MRI images of knee bone with deep learning techniques and five papers on those with machine learning techniques. Similarly, we studied 10 papers on CT scan images of knee bone with deep and machine learning techniques. Thus, our research fulfills the requirements of our first question.

### 5.2. Techniques or Methods Used

Deep learning is, overall, a developing approach in information investigation and has been named one of the 10 greatest advances of 2013. Deep learning is an improvement of artificial neural networks, comprising more layers that grant more significant levels of deliberation and improved expectations from the information. Until this point in time, it has been rising as the main AI device in the general imaging and computer vision spaces. Medical image examination groups all over the world are rapidly entering the field and applying CNNs and other deep learning approaches to a wide assortment of utilizations. Promising outcomes are developing. Most translations of medical images are performed by doctors; however, the picture understanding of people is restricted because of its subjectivity, huge variation across mediators, and weakness. Numerous symptomatic actions require an original search procedure to recognize variations from the norm and to evaluate estimations and changes after some time. Electronic instruments—explicitly, image examination and AI—are the key empowering influences to improve analysis, by encouraging recognizable proof of discoveries that require treatment and help the master’s work process. Among these devices, deep learning is quickly becoming the cutting edge approach, prompting improved precision. It has additionally opened up new countries to information examination with paces of progress not previously experienced.

Even though the term machine learning is generally recent, the ideas of machine learning have been applied to clinical imaging for quite a long time, maybe most eminently in the areas of computer-aided diagnosis (CAD) and useful brain mapping. Our objectives are to familiarize examination with some cutting edge strategies that are presently staples in the machine learning field and to delineate how these methods can be utilized in different manners in clinical imaging.

We designated deep learning and machine learning techniques to study knee bone images. Deep learning and machine learning techniques are given below in Table 15, which were used for this SLR.

### 5.3. Failure or Success Ratio

The artificial neural network (ANN), a machine learning procedure derived from the human neuronal neurotransmitter framework, was presented during the 1950s. In any case, the ANN has recently been restricted in its capacity to tackle genuine issues, because of the disappearing inclination and overfitting issues with preparing deep architecture, absence of registering power, and, fundamentally, the lack of adequate information to prepare the computer framework. Enthusiasm for this idea has recently remerged because of the accessibility of enormous amounts of information, upgraded registering power with current designs, preparing units, and novel calculations for preparing the profound neural system. Ongoing investigations into this innovation propose that it is conceivable for it to perform better than people in some visual and sound-related acknowledgment assignments, which may predict its applications in medicine and medical services, particularly in clinical imaging, working within a reasonable time-frame. This review article offers points of view on the history, improvement, and utilization of deep learning and innovation, especially concerning its applications in clinical imaging.

The purpose of this question was to determine the failure and success ratio of this SLR. We checked whether our research either completed successfully or failed. Thus, according to the results mentioned in Section 6, it is obvious that our SLR has been successful. It gave us a clear indication that there are a lot of techniques in a machine and deep learning that can provide the most accurate results for knee bone images that we get from X-ray, CT scan, or MRI reports.

### 5.4. Best Technique

For deep learning in radiology to succeed, it must be noted that well-explained huge informational indexes are required for deep systems that are mind-boggling; computer programming and equipment are developing continually, and inconspcious contrasts between malady states are harder to see than contrasts between regular items. Later on, machine learning in radiology will be required to be generously used in the clinic along with the imaging assessments being routinely performed in clinical work, providing the chance to improve choices and help in clinical picture understanding. The term of note is choice help, demonstrating that computers will expand human dynamics, making it progressively viable and proficient. The clinical effect of having PCs in routine clinical practice may permit radiologists to additionally incorporate their insights with their clinical associates in other clinical claims to fame and consider accurate medication.

We compared our study with deep learning and machine learning techniques; all techniques provide good accuracy for knee bone images. The accuracy of X-ray, CT scan, and MRI images with deep and machine learning techniques are mentioned in Table 16, below.

The results show that deep learning techniques and methodologies provide more accuracy for X-ray, MRI, and CT scan images of knee bones to diagnose diseases. Meanwhile, machine learning provides good accuracy but less than deep learning.

## 6. Conclusions and Forthcoming Work

The objective of this research was to conduct a comparative “systematic literature review” on MRI, CT scan, and X-ray images of knee bone by using deep learning techniques. Thirty-two papers associated with deep learning and machine learning approaches were reviewed in this research, which provide the accuracy mentioned in Table 17 and Table 18, respectively.

Let us discuss the outcomes of each research paper used for this research to finalize our results. The following points describe the outcomes of each paper:

**S1:** This paper is about deep learning techniques with X-ray images of the knee bone disease osteoarthritis. The authors showed a novel methodology for naturally diagnosing and reviewing knee OA from X-rays. It can help patients experiencing knee pain to obtain a quicker finding. Their technique accomplishes the best multi-class grouping results, despite having an alternate testing set: a normal multi-class precision of 66.71%, radiographical OA AUC of 0.93, quadratic weighted Kappa of 0.83, and MSE of 0.48. It may be possible to contrast this with normal human understandings. A total of 3000 subjects from the Osteoarthritis Initiative dataset was used in their research.

**S2:** This paper covers the knee bone tumor X-rays with deep learning techniques. The ImageNet dataset was used for this paper. In this paper, the researchers present a successful end-to-end deep learning model for knee bone tumor arrangement. In future work, the researchers will grow the present model to confine the tumor’s position, which may altogether support clinical treatment. The proposed new model uses semi-supervised ensemble Wnet (SSEW) in their research. They used three models—InceptionV3, ResNet50, and MobileNet. The accuracy results for the proposed model (SSEW) are 97.64 for Two-Class, 79.23 for “79.23”, and 80.31 for Five-Class, respectively.

**S3:** This paper is about studying the osteoarthritis of knee bones via X-rays with deep learning techniques. The Kellgren–Lawrence classification model is used for research. The accuracy of this model is, for moderate OA, 91.5%; for minimal OA, 80.4%; and for doubtful OA, 57%.

**S4:** This paper is related to X-ray images of knee bone osteoarthritis with deep learning techniques. This paper researched a few new techniques for the programmed measurement of knee OA seriousness, utilizing the CNN model. In the future, there is a plan to increase the accuracy of the CNN model of the knee joint by applying more techniques. The accuracy of this technique was 94.2%.

**S5:** The researchers worked on knee osteoarthritis in X-ray images of knee bone. The active contour segmentation technique was used for this research. A total of 200 X-ray images were used as the dataset. The accuracy of this technique was 87.92%.

**S6:** In this paper, X-ray images of osteoarthritis patients were used with deep learning techniques. Two algorithms were used, which are the K-nearest Neighbor Algorithm (KNN) and Supports Vector Machine (SVM). The accuracy results for these techniques were, for normal images, KNN = 100% and SVM = 79% and for abnormal images, KNN = 100% and SVM = 100%.

**S7:** This research paper is about MRI images of knee bones for the detection of knee bone diseases from MRI. Online MRI datasets were used for this technique. Scale Space Local Binary Pattern (LBP) feature extraction was used for this research. The accuracy attained in this research was 96.1%.

**S8:** In this paper, the convolutional neural network (CNN) was used for MRI images with deep learning techniques. The MICCAI dataset was used to accomplish this research. In this paper, the researchers built up a knee ligament division calculation from a high goal MR volume utilizing a novel completely 3D Convolutional Neural Network (CNN). The accuracy of this technique was 87.7%. The accuracy of the proposed system will be improved in the future.

**S9:** In this paper, 3D MRI datasets SK10, Dataset OAI Imorphics, and dataset OAI ZIP were used for MRI images of knee bones with deep learning techniques. The models used for this research were the 3D CNN and SSM. The accuracies of these models were for the 3D MRI datasets SK110, 75.73%; for the dataset OAI Imporphics, 90.4%; and for the dataset OAI ZIb, 98.5%.

**S10:** In this research 3D MRI dataset images were used for knee bone MRI reports. The researchers of this research created and assessed a programmed 3D deformable methodology for knee MRI having force inhomogeneity. The exploratory outcomes showed that their methodology accomplished 95% Dice, 93% SENS, and 99% SPEC in the volume assessment but an ASSD of 1.17 mm and RMSD of 2.01mm in the surface assessment.

**S11:** In this research, the Convolutional Neutral Network (CNN) model was used for 175 patient MRI datasets for knee bones. The accuracy attained in this research was 91%.

**S12:** This research is on CT scan image reports on knee bone. It is a systematic literature review. In this research, the datasets of CT scan, and some of MRIs, are used. Their results show that 95% accuracy can be achieved.

**S13:** In this study, 2D CT scan images were used for knee joint reconstruction. The CT scan data of multiple patients were used for this research. The researchers of this research used a convolutional neural network for all knee bone diseases. A dataset of 21 abdominal CT scans images was used to accomplish this research. The accuracy obtained from the research was 93%.

**S14:** The CT scan data of multiple patients was used by using different deep learning models. The technique models used to accomplish this research were Finite element (FE), Sobel Operator, Laplacian of Gaussian operator, and Canny edge detection. The researchers claim that 83% accuracy can be achieved for CT scan images of the knee by using these techniques.

**S15:** The researchers of this research used the CT scan images of 59 patients by using the CNN technique for sclerotic metastasis of knee bone CT scans. The researchers created a two-layered coarse-to-fine course structure to initially working with an exceptionally delicate hopeful age framework with the greatest affectability of ∼92% but with a high FP level (∼50 per understanding). The average accuracy gain with this technique was 70% to 80%.

**S16:** In this study, the researchers used the 3D image datasets of knee bone CT scans. The convolutional neural network was used for their research. In this paper, the researchers proposed a novel structure to determine seven anatomical milestones of the distal femur bone. They attained 80% accuracy in this research.

**S17:** In this study, the researchers worked on MRI images of Gaucher’s patients’ reports using machine learning techniques. The technique that was used to accomplish this research is the General Linear Model (GLM). This study’s motivation was to build up a strategy to measure the seriousness of bone ailments in type 1 GD patients and to differentiate between various GD genotypes and between GD patients and healthy people. The accuracy obtained with this technique for MRI images of knee bones was 70%.

**S18:** In this study, the researchers performed work on MRI images of osteoarthritis and osteochondral patients. The machine learning technique used for this study was Pattern Recognition and Multivariable regression (WND-CHARM). WNDCHRM might be an effective technique for the grouping of ligament MRIs. A dataset of 35 MRI images was used to accomplish this study. The accuracy obtained by this technique for MRI images of knee bones was 86%.

**S19:** In this study, the researchers studied the programmed characterization of finished tissues in 3D MRI. This research used the complex magnetic resonance signal, which provides segmented information. This research is good for the feature extraction of knee bone for all diseases. In this study, the Support Vector Machine (SVM) was used by using a dataset of 18 MRI images. A 91% accuracy was obtained by using this technique.

**S20:** In this paper, 36 BNC samples and 40 control samples of osteoarthritis patients’ MRI images were used. A multivariable Support Vector machine was used for this study. The researchers examined whether multivariate help vector machine examination would allow enhanced tissue portrayal. SVM examination performed utilizing certain parameter combinations displayed especially ideal grouping properties. The outcomes show the capacity of multivariate examination to incredibly increase the MRI appraisal of ligament network status in essential scientific studies. The research proves that 97% accuracy can be achieved for MRI images of knee bones using the SVM technique.

**S21:** In this research, the natural language processing framework was prepared, in an attempt to master ordered knee MRI reports from two noteworthy human services associations. The researchers assessed the execution of the framework both inside and across associations. The Support Vector Machine was used to accomplish this study. The results of this research prove that 95% accuracy can be achieved by using this technique for knee MRI reports.

**S22:** This paper tried to distinguish the edges of restorative pictures, especially knee osteoarthritis pictures in various basic states utilizing the Sobel administrator and proposed developed Sobel calculation. The proposed algorithm is very good for blurred and distorted images. The researchers used the dataset of X-ray images of osteoarthritis patients by using Sobel Edge Detector Operator. Their research concludes that 50% accuracy can be obtained by using this machine learning technique.

**S23:** In this paper, the researchers proposed a model-guided milestone limitation strategy for programmed ISR estimation. At first, the proposed technique required building a patella to demonstrate a factual patella shape and power data from preparatory information. They used 21 X-ray images for training patellae and 48 X-ray images as the validation data set. A component point extraction calculation was intended to determined the underlying model position naturally. The precision of the proposed technique was proved by both quantitative and subjective tests. Their research’s outcome is that 80% accuracy can be achieved by using this technique.

**S24**: This paper exhibited a researcher’s exploration of the recognition of bone breaks in X-ray images. A dataset of 432 images of the femur was used with the Support Vector Machine and Bayesian Classifier techniques. Their research concludes that 97.2% accuracy can be achieved through these machine learning techniques.

**S25:** In this study, the researchers used a dataset of 75,000 patients. They used the X-ray images of osteoarthritis patients by using the Bootstrap Aggregation technique. They achieved 97% accuracy for X-ray images of knee bone osteoarthritis.

**S27:** This investigation presented a robotized computer-aided diagnostic approach for the identification of OA, which utilizes a mix of standardizations given presciently, displaying MLR utilization and highlight extraction utilizing ICA. The classification rates of the proposed CAD are higher than those obtained in previous studies. They used 1024 X-ray images of osteoarthritis patients and used the Multivariate Linear Regression (MLR) technique. They achieved 82.98% accuracy.

**S28:** This research study performed a morphometrical comparison between CT scan databases of miniaturized objects by using the two-division procedure. The researchers used the Micro CT-Scan trabecular datasets by using Chan–Vese and Fixed Threshold techniques. They obtained 60% to 70% accuracy with these techniques.

**S29:** This study is related to CT scan images of osteoporosis, which is a knee bone disease. They used the Multidetector Computed Tomography (MDCT) dataset of CT scan images of knee bones. They obtained 98% accuracy.

**S30:** The goal of this study was to utilize fluoroscopy to precisely determine three-dimensional (3D) structure. They used 10 knees-experienced patterns of 3D CT scan images by using the helical axis of the model. They obtained 90% accuracy.

**S31:** The primary objective of this study was to assemble a limited component, demonstrate phenomena for trabecular bone from small scale figured tomography (miniaturized scale CT) pictures, and concentrate on the flexible properties of bone. They used seven samples of pig femur bone CT scan images by using the finite element model. They obtained 65% accuracy by using this machine learning technique.

**S32:** In this examination, a few morphological parameters were estimated at the same time for each example as opposed to simply concentrating on a couple of parameters. The researchers of this study used the Finite Element Model (FME) on the CT scan images of porcine trabecular bones. They obtained 70% accuracy with the FME for CT scan images.

We determined our results by calculating the average of each image type, i.e., to determine the accuracy of X-ray images for deep learning, we summed up the accuracy of each X-ray paper and divided the sum by the total number of X-ray papers. In our study, there are six X-ray papers for deep learning, so we calculated it by dividing the accuracy sum by six. Similarly, we calculated the total accuracy for the MRI and CT scan image types.

After obtaining the results for all the papers, we determined that deep learning methodologies provide more accuracy for MRI, CT scan, and X-ray images than machine learning techniques. While machine learning techniques provide less accuracy, we can conclude by saying that deep learning techniques are best for X-ray, MRI, and CT scan images for knee bone diseases. Undoubtedly, techniques of deep and machine learning are both helpful in medical image processing, but it is the situation that decides which is the best technique to obtain the best accuracy.

As it is a comparative systematic literature review, we conclude by finding and calculating each study’s accuracy.

The explosion of computerized medical service information has prompted a flood of information-driven clinical exploration dependent on machine learning. As of late, as an influential strategy for big data, deep learning and information retrieval have increased a focal situation in machine learning circles for incredible points of interest in inclusion portrayal and example acknowledgment. Many articles present a far-reaching outline of studies that utilize big data and information retrieval strategies to manage clinical information. Initially, because of the investigation of the attributes of clinical information, different kinds of clinical information (e.g., clinical pictures, clinical notes, lab results, indispensable signs, and segment informatics) are discussed, and the subtleties of some open clinical datasets are mentioned. Furthermore, a short review of normal deep learning models and their qualities is performed. At that point, considering the wide scope of clinical exploration and the considerable variety of information types, a few big data applications for clinical information are shown: assisted conclusions, anticipation, early admonition, and different errands. Even though there are difficulties in applying deep learning procedures to clinical information, it is as of yet advantageous to anticipate a promising future for deep learning applications in clinical big data toward precision medicine [49].

Our research is still in progress; as many studies are performed in the medical field by using big data and information retrieval techniques, we have a plan to review big data and information retrieval techniques for this literature review to make this study more qualified.

Table A1 contains the citation’s references of selected studies. By using this table, reader can find the reference of selected studies from bibliography.

## Figures and Tables

**Table 1:** Research questions.

QID	Research Questions	Motivation
RQ1	Which sort of knee bone images are used for medical image processing in this literature review?	To understand the focus of knee bone images by using the different methodologies.
RQ2	Which techniques or methods are used to study the selected knee bone images?	To identify the methods or technologies for identifying the disease from knee bone images.
RQ3	What are the failure and success ratios?	To identify which techniques are best and worst for medical image processing.
RQ4	Which method or technology is best for which image type?	To identify which technique provides more accuracy for X-rays, MRI, and CT scans.

**Table 2:** String search.

Population	Intervention
(Knee Bone) AND (Deep Learn *)	(MRI OR CT-Scan OR X-ray OR Oesth * OR Convolutional Neural Netw * OR Disease * OR Issues OR Challenges OR Accura *)
(Knee Bone) AND (Machine Learn *)	(MRI OR CT-Scan OR X-ray OR Oesth * OR Convolutional Neural Netw * OR Disease * OR Issues OR Challenges OR Accura *)

**Table 3:** Study inclusion criteria.

Methodology/Techniques	Image Type	#
**Deep Learning**	X-ray	6
	MRI	5
	CT-Scan	5
**Machine Learning**	X-ray	6
	MRI	5
	CT-Scan	5

**Table 4:** Quality criteria for primary study selection.

Type	Definition
Internal Validity	The point of the investigation, setting, and suppositions are introduced in the examination.
External Validity	The discoveries ought to be sufficiently genuine to be connected in the industry or the scholarly community.
Construct Validity	The connection between the research questions used to quantify/assess and the results are well characterized.
Conclusion Validity	The study and ends are consistent with the RQs and obtained from experimental/hypothetical investigation.

**Table 5:** Data extraction form.

Purpose	Metadata
**General Information**	Tables 9 and 10 provide the information extraction frame that is utilized for all the chosen essential investigations to do a top-to-bottom study. It describes the author’s name of the paper, published year, image type, model, data set, disease, and accuracy of the proposed techniques.
**Specific Information**	Tables 11 and 12 demonstrate the work of the researchers that they have done by using different methodologies in their research papers, the prediction of their future work, and the advantages of their research.
**Explicit Information**	Tables 13 and 14 determine the gaps in the review articles on deep learning and machine learning papers.

**Table 6:** Quality assessment of selected research papers on deep learning.

SP#	QA1	QA2	QA3	Score	Score %
SP1	A	D	A	2	66.67
SP2	S	A	D	1.5	50
SP3	A	A	A	3	100
SP4	A	S	A	2.5	83.33
SP5	D	A	S	1.5	50
SP6	A	A	A	3	100
SP7	S	D	S	1	33.33
SP8	A	A	A	3	100
SP9	S	A	D	1.5	50
SP10	A	A	A	3	100
SP11	D	S	D	0.5	16.66
SP12	A	D	A	2	66.67
SP13	D	S	A	1.5	50
SP14	A	A	A	3	100
SP15	S	A	A	2.5	83.33
SP16	S	A	A	2.5	83.33
Total	10.5	11.5	11	34	70.83

**Table 7:** Quality assessment of selected research papers on machine learning.

SP#	QA1	QA2	QA3	Score	Score %
SP17	D	S	A	1.5	50
SP18	A	S	A	2.5	83.33
SP19	A	A	S	2.5	83.33
SP20	S	A	S	2	66.67
SP21	D	A	A	2	66.67
SP22	S	A	A	2.5	83.33
SP23	A	D	S	1.5	50
SP24	A	A	A	3	100
SP25	D	A	D	1	33.33
SP26	A	A	A	3	100
SP27	A	A	A	3	100
SP28	A	S	A	2.5	83.33
SP29	A	D	A	2	66.67
SP30	A	A	A	3	100
SP31	D	A	A	2	66.67
SP32	S	A	A	2.5	83.33
Total	10.5	12.5	13.5	36.5	76

**Table 8:** Citation of selected Deep Learning and Machine Learning Papers.

S#	Citation	Database	S#	Citation	Database
S1	8	Science Direct	S17	2	Science Direct
S2	6	IEEE	S18	13	Science Direct
S3	110	IEEE	S19	48	Science Direct
S4	29	IEEE	S20	27	Google Scholar
S5	7	Google Scholar	S21	9	Google Scholar
S6	0	Springer	S22	8	IEEE Xplore
S7	0	ACM	S23	7	Google Scholar
S8	1	IEEE	S24	51	Google Scholar
S9	1	Science Direct	S25	5	Springer
S10	0	ACM	S26	212	Science Direct
S11	9	Google Scholar	S27	0	Science Direct
S12	2	Science Direct	S28	1	IEEE Xplore
S13	0	Google Scholar	S29	1	Google Scholar
S14	2	Google Scholar	S30	245	Science Direct
S15	17	Springer	S31	0	Google Scholar
S16	22	IEEE	S32	0	Google Scholar

**Table 9:** Extracted data for knee bone-related MRI, CT scans, and X-rays using deep learning techniques.

#	Author	Year	Image Type	Data Set	Model	Disease	Accuracy
**S1**	“A. Tiulpin, J. et al.”	2018	X-ray	3000 subjects from the Osteoarthritis Initiative dataset.	Deep Siamese Convolutional Neural Network (CNN)	Osteoarthritis	Accuracy = 66.71%, ROC curve = 0.93, AUC = 0.93, MSE = 0.48
**S2**	“H. -J. Yang et al.”	2018	X-ray	ImageNet dataset	Proposed new model semi-supervised ensemble Wnet (SSEW)	Knee Bone Tumor	Two-class = 97.64% Fourth-class = 79.23% Five-class = 80.31%
**S3**	“L. Shamir et al.”	2009	X-ray	350 images of X-ray	Kellgren–Lawrence classification	Osteoarthritis	For moderate OA = 91.5%; for minimal OA = 80.4%; for doubtful OA = 57%
**S4**	“J. Antony et al.”	2016	X-ray	Bilateral PA fixed Flexion knee X-ray images	Convolutional Neural Networks (CNN)	Knee Osteoarthritis	94.2%
**S5**	“S. S. Gornale et al.”	2016	X-ray	200 datasets of knee X-ray images	The active contour segmentation technique	Osteoarthritis	87.92%
**S6**	“Hegadi et al.”	2019	X-ray	X-ray images of OA patients	K-Nearest Neighbor Algorithm (KNN) Support Vector Machine (SVM)	Osteoarthritis	For normal images (KNN = 100%, SVM = 79%) For abnormal images (KNN = 100%, SVM = 100%)
**S7**	“J. Mun et al.”	2018	MRI	Online MRI dataset	Scale Space Local Binary Pattern (LBP) feature extraction, SVM	Detection of knee bone from MRI Images	Accuracy = 96.1% MCC rate = 88.26%
**S8**	“S. Vishwanatn et al.”	2018	MRI	MICCAI datasets	CONVOLUTIONAL NEURAL NETWORKS (CNN)	Osteoarthritis	87.7%
**S9**	“F. Ambellan et al.”	2019	MRI	3D MRI datasets SKI10 Dataset OAI Imorphics Dataset OAI ZIB	3D CNNs, SSMs	Osteoarthritis	3D MRI datasets SKI10 = 75.73%, Dataset OAI Imorphics = 90.4%, Dataset OAI ZIB = 98.5%
**S10**	“D. Kim et al.”	2018	3D MRI	MRI datasets of knee	3D deformable approach	Osteoarthritis	DICE = 0.951, SENS = 0.927, SPEC = 0.999, ASSD = 1.16, RMSD = 2.01
**S11**	“F. Liu et al.”	2018	MRI	MRI datasets of 175 patients	Convolutional Neural Networks (CNN)	Cartilage Lesion	91%
**S12**	“X. -D. Wu et al.”	2017	CT-Scan images and MRI images	CT scan and MRI data collected from multiple types of research	Systematic literature Review	Osteoarthritis	95%
**S13**	“F. La Rosa et al.”	2019	2D CT scan images	Dataset of 21 abdominal CT scan images	Convolutional Neural Networks (CNN)	Applicable for all knee bone diseases for image segmentation	93%
**S14**	“S. -W. Jang et al.”	2014	CT scan images	CT scan data for multiple patients	“Finite Element (FE)”, “Sobel operator”, “Laplacian of Gaussian operator”, “Canny edge detection”	Knee joint Reconstruction	83.23%
**S15**	“H. R. Roth et al.”	2014	CT scan images	CT images of 59 patients	Deep, CNN	Sclerotic metastases	70–80%
**S16**	“D. Yang et al.”	2015	3D images	3D image data for knee joints	CNN	Detection on Distal Femur Surface	80%

ROC = receiver operating characteristic curve, AUC = Area under the ROC Curve, OAI = Osteoarthritis Initiative, ZIB = zero-inflated binomial, SSMs = Statistical Shape Models, DICE = Diverse Counterfactual Explanations, SPEC = Standard Performance Evaluation Corporation, RMSD = root-mean-square deviation.

**Table 10:** Extracted data for knee bone-related MRI, CT scans, and X-rays using machine learning techniques.

#	Author	Year	Image Type	Data Set	Model	Disease	Accuracy
S17	“G. B. Sharma et al.”	2016	MRI images	Tested reports of Gaucher patients	General Linear Model (GLM)	Gaucher Disease	70%
S18	“B. G. Ashinsky et al.”	2015	MRI images	35 MRI images	Pattern recognition and multivariable regression (WND-CHARM)	Osteoarthritis Osteochondral	86%
S19	“P. Bourgeat et al.”	2007	3D MRI	Dataset of 18 images	Support Vector Machine (SVM)	Feature extraction of knee bone for all diseases	91%
S20	“P. Lin et al.”	2018	MRI	(1) 36 BNC samples (2) 40 control samples	Multivariate Support Vector Machine	Osteoarthritis	97%
S21	“S. Hassanpour et al.”	2017	MRI Images	Knee MRI Reports	Support Vector Machine	Diagnostic yield of knee bone reports	95%
S22	“S. Zahurul et al.”	2010	X-ray images	X-ray images of patients	Sobel Edge Detector Operator	Osteoarthritis	50%
S23	“H. -C. Chen et al.”	2010	X-ray images	(1) 21 images for training patella (2) 48 images as the validation set	Registration-assisted active shape model	Lateral knee	80%
S24	“S. E. Lim et al.”	2004	X-ray images	432 images of the femur	Support Vector Machine and Bayesian Classifier	Osteoporosis	97.2%
S25	“C. Kruse et al.”	2017	X-ray	75,000 patients’ data	Bootstrap Aggregation	Osteoarthritis	95%
S26	“W. A. Hoff et al.”	1998	3D X-ray images	24 X-ray images	Computer-aided Design (CAD)	Femoral tibia	50%
S27	“Brahim et al.”	2019	X-ray Images	1024 X-ray images	Multivariate Linear Regression (MLR)	Osteoporosis	82.98%
S28	“F.Demenegas et al.”	2011	CT scan images	Micro CT trabecular datasets	Chan–Vese and Fixed Threshold	For all diseases of knee bone	60% to70%
S29	“C. Chen”	2014	CT scan images	Multidetector Computed Tomography (MDCT) dataset	Finite Element Method	Osteoporosis	98%
S30	“D. A. Dennis et al.”	2005	3D CT scan images	10 knees, experienced patterns	The helical axis of the model	Anterior cruciate ligament-deficient	90%
S31	“O. Jin”	2015	CT images	Seven samples of pig femur bone	Finite element model	Non-specific study, elastic study of bone	65%
S32	“D. Yang et al.”	2013	CT images	CT scan images of patients	Finite Element Model (FME)	Porcine Trabecular Bone	70%

**Table 11:** Extracted data from papers including contributions, predictions, and advantages of related deep learning techniques.

#	Contribution	Prediction	Advantage
S1	In the present examination, the authors showed a novel methodology for naturally diagnosing and reviewing knee OA from plain radiographs. Rather than past examinations, their model uses explicit highlights significant for the ailment, ones that are practically identical to the ones utilized in clinical practices(e.g., bone shape, joint space, etc.). Besides, considering the recently described methodologies, their technique accomplishes the best multi-class grouping results, despite having an alternate testing set: a normal multi-class precision of 66.71%, AUC of 0.93 for radiographical OA detection, quadratic weighted Kappa of 0.83, and MSE of 0.48. This can be contrasted with normal human understandings. [4]	The considerable description of their model unmistakably shows that it adapts progressive neighborhood inclusions that feature genuine important radiological discoveries. The most likely reason is that they forced space learning limitations (earlier anatomical information) in the system’s design, in this way, driving it to learn just the highlights identified with the radiographical discoveries, for example, osteophytes, bone distortion, and joint-dividing narrowing, which are altogether used to review the picture as indicated by the KL scale. They additionally report another clinically applicable outcome, the likelihood appropriation of the KL reviews of the pictures. These data come genetically from the system’s design and can be utilized as another wellspring of advantageous analytic data. For instance, if the model is not sure about the expectation (prediction), this is found in the distributions.	It was prepared exclusively with the MOST dataset and tried with the OAI dataset. The principal preferred aspect of this current study’s structure was the exhibition of the model’s capacity to learn significant OA highlights that are transferable across various datasets. This demonstrates that their strategy is strong for various phenomena and information-obtaining settings. It can help patients experiencing knee pain to obtain a quicker finding. Social insurance, by and large, will profit by a decrease in the expenses of routine work.
S2	In this paper, the researchers provide a successful end-to-end deep learning model for knee bone tumor assessment. The proposed model considers the quality of a mix of supervised and unsupervised methods of detecting significant patterns for recognizing ordinary/anomalous bone and characterizing what kind of bone a tumor is. Besides, they apply the troupe system to improve the general execution. The test results demonstrate that their proposed model beats the notable existing models. [19]	In future work, the researchers will grow the present model to determine the tumor’s position, which may altogether support clinical treatment.	The researcher’s proposed model SSEW utilizes determines how to produce the more auxiliary highlights unsurpervised, which not just decreases the computational expense but also provides more data for characterization. Besides, they apply the joint system to improve precision in terms of bone tumor characterization.
S3	In this paper, the authors depicted a computerized methodology for the location of OA using knee X-rays. Without an exact technique for OA analysis, the physically grouped KL review was utilized. The order is not performed such that endeavors to emulate the human grouping, however, depends on an information-driven methodology utilizing physically arranged X-beams of various KL grades, speaking to various phases of OA seriousness. The test results suggest that over 95% of moderate OA cases were accurately separated from ordinary cases, with a false positive rate of ∼12.5%. The order exactness for separating negligible OA from ordinary cases was ∼80%, and the discovery of dicey OA cases was far less persuasive. [20]	Future endeavors for enhancing the location precision for suspicious OA will incorporate the joining of important clinical data, for example, history of knee damage, body weight, and knee arrangement points; furthermore, they will likewise utilize more X-ray tests, as they are accessible.	This study was performed in the unique situation of a longitudinal aging study that will improve the examination of imaging information not exclusively for clinical OA issues such as pain but also for physiological estimates to apply to aging body issues that may add to OA seriousness.
S4	This paper researched a few new techniques for the programmed measurement of knee OA seriousness utilizing the CNN model. This is increasingly exact and, furthermore, quicker than layout coordination. Their underlying way of determining the knee OA seriousness utilized highlights removed from pre-prepared CNNs. [21]	In the future, there is a plan to increase the accuracy of the CNN model of the knee joint by applying more techniques.	Past examinations have surveyed their calculations utilizing parallel and, furthermore, multi-class order measurements. This methodology was prepared to utilize relapse misfortune with the goal that mistakes are punished to the extent of their seriousness, creating more precise expectations. This methodology likewise has the advantageous property that the expectations can fall between evaluations, which accords with a nonstop infection situation.
S5	Their methodology consists of 5 major steps: Image Acquisition, Image Pre-processing, Image Segmentation, Image Enhancement, and Feature Extraction, and their experimental results gained by applying the following 7 steps of their proposed active contour algorithm: The first step is pre-handling, i.e., clamor removal, picture handling, resizing, etc., and conversion to dim scale. The second step is normalizing the blurred scale picture to measure 250x250 for further investigation. The third step is segmentation, which utilizes the active shape calculation. The fourth step is to divide picture, which is improved utilizing a contrast change procedure to enable further understanding. In the fifth step, the diverse highlights are registered as shape highlights, statistical highlights, first-four minutes, Haralick highlights, and texture examination highlights. In the sixth step and lastly, 40% preparation and 60% testing is done on the acquired rundown of highlights utilizing a Random Forest classifier. The seventh step is the end of the calculations and results. [22]	In the future, an innovation or process should be produced that is related to osteoarthritic pain and clinical side effects, for example, regardless of whether the side effects are identified with joint tissue, neuropathic pain, solid pain, etc. This may help in obtaining a great arrangement rate.	A knee X-ray picture is especially inclined to undesirable bends, which may be caused by the issue breaking down the bone structures. To overcome these issues, researchers have utilized a semi-computerized strategy that represents a speedy and productive technique for dissecting the variations from the norm and issues related to the bone structures.
S6	Their proposed technique could order both ordinary and anomalous pictures accurately utilizing the KNN and cubic SVM classifier. The grouping rate for typical pictures utilizing the KNN classifier is 100%; however, for the SVM, it is 79%. For irregular pictures, the KNN and, furthermore, SVM give 100% precision. The general characterization exactness of the SVM classifier is 89%. These two grouping strategies show fourfold cross-approval. [23]	In specific cases, the calculation neglects to determine the focal piece of the synovial cavity area, which might be considered for future upgrades.	The proposed method provides more accuracy than the existing algorithm.
S7	In this research, the authors utilized SSLBP inclusion extraction, a variation of the neighborhood paired example, to prepare and order the pre-handled MRI images utilizing SVM. The exploratory outcome demonstrated that their methodology had higher ACC and MCC values than fluffy c-implies and, furthermore, deep component extraction techniques. Exact knee bone recognition through the proposed model would be an essential aid for the improvement of a completely self-sufficient careful framework. [24]	Researchers have no plan to do more research on this topic	The after-effects of their proposed methodology show higher ACC and MCC values than force-based strategies, particularly for MCC results. These exploratory outcomes demonstrate that the SSLBP highlight extraction connected to the SVM is better than the existing force-based picture-preparing instruments, for example, fluffy c-implies calculations. Additionally, the SSLBP highlights which things separated from their instincts in the proposed strategy beat the separated highlights from the DNN with ImageNet.
S8	In this paper, the researchers built up a knee ligament division calculation from a high goal Machine Learning MR volume utilizing a novel, completely 3D Convolutional Neural Network (CNN). This is, to our knowledge, the main programmed ligament division strategy utilizing 3D CNNs. The proposed calculation performed superiorly to the best in the class calculation in the MICCAI SKI10 open test. They additionally connected their proposed calculation to another comparative MR differentiate (DESS) given by the Osteoarthritis Initiative (OAI) for OA evaluation and introduced enhanced division correctness. An initial subjective appraisal of the division results outwardly delineates ligament problems from longitudinal knee MR information. [25]	The accuracy of the proposed system will be further improved in the future.	Researchers have demonstrated that u-net performed superiorly to the current cutting edge technique within clinically satisfactory runtimes. Their Dice score measures fluctuate between 78.5% and 85.7% for different ligament surfaces for information goals of 1 × 1 × 1. 5 mm^3^. These Dice scores are near the revealed mean inter-observer reproducibility of 87.7%. They accept that the proposed strategies will have enhanced exactness and facilitate the programmed quantitative assessment of knee ligament morphology for the appropriation of the quantitative MRI methods for OA in routine clinical practice.
S9	The researchers present a strategy for the mechanized division of knee bones and ligaments from MRI that consolidates the earlier information of anatomical shape with Convolutional Neural Networks (CNNs). The proposed approach fuses 3D Statistical Shape Models (SSMs) just as 2D and 3D CNNs to accomplish a stronger and exact division of even very unusual knee structures. The shape models and neural systems utilized are prepared to utilize information from the Osteoarthritis Initiative (OAI) and the MICCAI‘s amazing test “Division of Knee Images 2010” (SKI10), separately. They assess their strategy on 40 approval and 50 accommodation datasets from the SKI10 challenge. Unexpectedly, an exactness proportionate to the between-eyewitness changeability of human perusal is accomplished in this test. Additionally, the nature of the proposed strategy is completely assessed utilizing different measures for information from the OAI—for example, 507 manual divisions of bone and ligaments, and 88 extra manual divisions of the ligaments. Their technique yields sub-voxel exactness for both OAI datasets. They make the 507 manual divisions, like their exploratory setup, freely accessible to additionally help exploration in the field of therapeutic picture division. Taking everything into account, consolidating confined arrangements through CNNs with measurable anatomical learning using SSMs results in a best-in-class division strategy for knee bones and ligaments from MRI information. [26]	Later on, a more upfront assortment of SSMs and CNNs may improve the advantages of shape information and offer the capability to the CNN of preparing and also differentiating low-scale datasets. To help this advancement, the manual divisions made by experienced clients at the Zeus 570 Institute Berlin are made freely accessible as a feature of this distribution.	In the researcher’s work, the robotized strategy lessens the time needed for an exact division of knee bones and 505 ligaments by a factor of six compared with manual divisions by an accomplished user.
S10	The researchers of this research created and assessed a programmed 3D deformable methodology in knee MRI having force inhomogeneity. They showed that the underlying point can be resolved dependomg on the histogram with earlier learning. It is additionally striking that no preparation stage is required, dissimilarly to in other custom deformable models, for example, ASM, AAM, and map book-based models. Exploratory outcomes showed that their methodology accomplishes 95% Dice, 93% SENS, and 99% SPEC in the volume assessment but an ASSD of 1.17mm and RMSD of 2.01mm in the surface assessment. [5]	Later on, they have plans to, for the most part, improve the after-effects of surface in the 3D division. Additionally, they will assess other arrangement types of X-rays to demonstrate their suitability for application to the therapeutic field.	The outcome demonstrates that their proposed methodology is valuable for performing basic and precise bone division for knee bone analysis.
S11	A completely robotized deep learning-based ligament sore location framework was produced by utilizing division and, furthermore, arrangement convolutional neural systems (CNNs). Fat-smothered T2-weighted quick turn reverberate MRI informational collections of the knees of 175 patients with knee issues were reflectively broken down by utilizing the deep learning strategy. The reference standard for preparing the CNN order was the elucidation given, by the cooperating prepared musculoskeletal radiologist, of the nearness or nonappearance of a ligament sore in 17,395 little picture patches of the articular surfaces of the femur and tibia. Recipient working bend (ROC) examination and the k measurement were utilized to survey symptomatic execution and intraobserver understanding for identifying ligament sores for two individual assessments performed according to the ligament core location framework. [27]	Extra specialized improvement and approval work is expected to enhance the present ligament injury discovery framework. Upgrades to the indicative execution could be accomplished if the CNNs could assess various groupings with various tissue contrasts.	This investigation exhibited the possibility of utilizing a completely robotized deep learning-based ligament injury identification framework to assess the articular ligament of the knee joint with high demonstrative execution and great intraobserver understanding for distinguishing ligament degeneration and intense ligament damage.
S12	In this systematic literature review, six examinations with an aggregate of 336 knees met the qualification criteria, and the four preliminaries were incorporated into the meta-examination. Contrasted and MRI-based PSI frameworks and CT-based PSI frameworks were related to a higher anomaliy frequency of coronas in the general appendage arrangement. While there were no noteworthy contrasts in the coronal/sagittal arrangement of the femoral/tibia segment exceptions, the precise coronal blunders in the general appendage arrangement, the rakish mistakes in the femoral/tibia segments in the coronal plane, or the frequency of increases in the embedded sizes of the femoral/tibia segments were monitored. [28]	This meta-investigation proposed that MRI-based PSI frameworks are related to a lower occurrence of anomalies of coronas with general appendage arrangements, smaller rakish coronal phenomena with general appendage arrangements, and shorter activity times than CT-based PSI. Both CT-and MRI-based PSI have advantages and disadvantages and are powerless to differentiate between many issues; therefore, PSI requires constant enhancement to accomplish better clinical results than have previously been prescribed for routine use.	To the best of researchers‘ insight, this is the first orderly study and meta-investigation of planned relative preliminaries to thoroughly and efficiently look into the current accessible literature and discover that MRI-based PSI offers potential clinical points of interest compared with the CT-based PSI: CT-based PSI is related with a higher rate of coronal anomalies and large appendage arrangements, bigger coronal rakish blunders and large appendage arrangements, and longer activity time requirements.
S13	This work shows the credibility of the program’s bone division and characterization of CT scans utilizing a CNN. The model accomplishes a high Dice coefficient and ends up being very robust to Gaussian clamor. In any case, a few constraints and enhancements that would unquestionably expand the execution are distinguished. An enhanced yet comparative model could be helpful in a few clinical and examine applications for numerous therapeutic undertakings. [6]	Later on, an extremely intriguing improvement would comprise actualizing the system with 3D conventional layers. As the bones are 3D structures, the system would have the capacity to extricate more highlights and likely accomplish a higher division exactness. Particularly, the ribs, which present an extremely divided shape in transversal cuts, need to be better represented.	The proposed CNN approached in this research will improve the accuracy of bone segmentation.
S14	With the popularity of picture division to create a 3D display from CT information, enthusiasm for target appraisal for the 3D models acquired utilizing different division strategies has been expanding. [29]	Taking everything into account, the Canny edge identification and calculation indicated great execution in the results of 3 out of 5 tests, showing that it is ideal for the remaking of a 3D solid model as compared to other approaches used for this research.	The Canny edge detection algorithm is best for constructing a 3D solid model.
S15	The researchers structure a two-layered coarse-to-fine course structure to initially develop an exceptionally delicate and hopeful age framework with the greatest affectability of ∼92% but with a high FP level (∼50 per understanding). Areas of intrigue (ROI) for injury hopefuls are produced in this progression and capacity as a contribution to the second level. At the second level, we create N 2D sees, utilizing scale, arbitrary interpretations, and pivots as for every rouse centroid organization. These irregular perspectives are utilized to prepare a profound Convolutional Neural Network (CNN) classifier. In testing, the CNN is utilized to allocate singular probabilities for another arrangement of N irregular perspectives that are arrived at at the midpoint of every rouse to register at last, for each hopeful, the grouping likelihood. This second level acts as a profoundly specific procedure to dismiss troublesome false positives while preserving high sensitivity. We approve the methodology for the CT pictures of 59 patients (49 with sclerotic metastases and 10 typical controls). The proposed technique lessens the quantity of FP/Vol. From 4 to 1.2, 7 to 3, and 12 to 9.5 when looking at affectability rates of 60, 70, and 80% separately in testing. The Area-Under-the-Curve (AUC) is 0.834. The outcomes indicate marked enhancement compared to in past work. [30]	The mechanized identification of sclerotic metastases (bone sores) in Computed Tomography (CT) pictures can be an essential apparatus in clinical practice and research. The best-in-class strategies demonstrate execution with a 79% affectability or genuine positive (TP) rate, with 10 false-positives (FP) per volume.	The researchers results‘ showed that their proposed methodology provides more accuracy than previous work.
S16	In this paper, the researchers proposed a novel structure to determine seven anatomical milestones of the distal femur bone. Their methodology is programmed, and it consolidates both worldwide shape data and nearby work shapes. The precise confinement of the anatomical tourist spots on the distal femur bone in the 3D restorative pictures is vital for knee medical procedure arranging and biomechanics examination. Be that as it may, the milestone ID process is frequently led physically or by utilizing the embedded assistants, which is tedious and lacks precision. In this paper, a programmed restriction technique is proposed to determine the places of introductory geometric tourist spots on the femur surface in 3D MR pictures. Because of the outcomes from the convolutional neural system (CNN) classifiers and shape measurements, we utilize limited band diagram slice improvement to accomplish the 3D division of the femur surface. Eventually, the anatomical tourist spots are situated on the femur as indicated by the geometric signals from the surface work. Tests show that the proposed strategy is powerful, productive, and reliable for sectioning femurs and determining anatomical milestones. [31]	There are a few bearings for future research work. One conceivable bearing is to expand preparing fluctuation and perform course location for higher accuracy. This work could likewise be extended to determine other anatomical milestones for different bones (for example, tibia) or organs in restorative pictures with contrast modalities.	Their investigation adds to the useful utilization of the 3D restorative picture preparation by enhancing the exactness of milestone limitation.

**Table 12:** Extracted data from papers including the contributions, predictions and advantages of related Machine Learning Techniques.

#	Contribution	Prediction	Advantage
S17	In this research article, machine learning-based examination distinguished bone miniaturized scale structure changes that may clarify the pathogenesis of GD bone delicacy (Bone-GD Tablet is used in the treatment of Osteoarthritis), provide markers to evaluate the seriousness of bone defects, and provide a quantitative standard for scoring therapeutic interventions. Work is in progress to apply this method to deal with the study of bigger subtype test sizes and perform 3D small-scale design biomechanical examinations. [32]	Concerning the viability of the exhibited procedures, the researchers of this paper have a plan to more comprehensively examine the trabecular bone of various subgroups with coordinated controls, with the objective of distinguishing subgroups at more serious risk, as well as identifying treatment adequacy sooner.	This present study’s motivation was to build up a strategy to measure the seriousness of bone ailments in type 1 GD patients and to differentiate between various GD genotypes and between GD patients and healthy people.
S18	A way to deal with the MRI images of ligament debasement is proposed utilizing structure acknowledgment and multivariable relapse in which picture highlights from the MRIs of histologically scored human articular ligament plugs were processed utilizing weighted neighbor separation utilizing a compound chain of importance of calculations, related to morphology (WND-CHRM). The WND-CHRM technique was first connected to a few clinically accessible MRI scan types to perform a parallel classification of typical and osteoarthritis osteochondral plugs depending on the Osteoarthritis Research Society International (OARSI) histology framework. Additionally, the picture highlights registered from WND-CHRM were utilized to build up various straight least-squares relapse demonstrations for the order and expectation of OARSI scores for every ligament plug. [33]	Numerous direct least-squares relapse predictions effectively anticipated OARSI scores and arranged attachments with correctnesses as high as 86%.	WNDCHRM (is an open source utility for biological image analysis) might be an effective technique for the grouping of ligament MRIs.
S19	The researchers study the programmed characterization of finished tissues via 3D MRI. With an MRI flag structure, there is no need for unfolding. The additional data separated from the stage allow better division than just utilizing greatness highlights. A greatness increment is acquired by diminishing the number of pixels that should be characterized [7].	Forthcoming work will investigate different procedures to remove stage data without stage unwrapping and consider different organs and groupings where the stage can help division and investigation.	This research uses the complex magnetic resonance signal, which provides the segmented information.
S20	The researchers examined whether multivariate help vector machine examination would allow enhanced tissue portrayal. SVM examination was performed utilizing certain parameter combinations especially ideal for grouping properties. All in all, ordinarily, the parametric MRI appraisal of grid status in a degenerative ligament is restricted by the cover in parameter esteems between shifting degrees of debasement. [34]	The outcomes show the capacity of multivariate examination to incredibly improve the MRI appraisal of ligament network status for essential scientific studies.	Deprivation probabilities obtained from the SVM technique exhibited extraordinarily more grounded relationships with biochemical estimations than did singular MRI parameters.
S21	In this research, the natural language processing framework was prepared, to master ordered knee MRI reports from two noteworthy human services associations. Radiology reports were displayed in the preparation set as vectors, and a help vector machine structure was utilized to prepare the classifier. A different test set from every association was utilized to assess the execution of the framework [35].	The information strengthens the attainability of the multi-institutional characterization of radiological imaging content reports with a solitary machine learning classifier without requiring a foundation explicit preparation of information.	The researchers assessed the execution of the framework both inside and across associations.
S22	This method was utilized to distinguish the edges of restorative pictures, especially knee osteoarthritis pictures, in various basic states utilizing Sobel administrator and the proposed developed Sobel calculation. Furthermore, the actualized program is exceptionally productive at runtime and good at identifying edges. Moreover, the program has overcome numerous weaknesses, for example, obscuring and commotion affectability [36].	The entire program will be actualized in a chip base to enhance execution time for high-caliber and little-differentiable pictures.	The proposed algorithm is very good for blurred and distorted images.
S23	In this paper, the researchers have proposed a model-guided milestone limitation strategy for programmed ISR estimation. At first, the proposed technique required building a patella to demonstrate factual patella shapes and power data from preparatory information. A component point extraction calculation was used to determine the underlying model position naturally [37].	Later on, the proposed strategy can be utilized to examine the impacts of subjects‘ ages, races, life propensities, or work types on the typical scope of ISR. On the off-chance that there are any all-encompassing studies or applications that require more prominent exactness or proficiency of confinement, we may embrace the multi-goals system for enhancing the framework execution.	The precision of the proposed technique was confirmed by both quantitative and subjective tests. The concordance between the results and physically estimated ISRs was effectively proved by a high relationship coefficient.
S24	This paper exhibits a researcher’s exploration of the recognition of bone breaks in X-ray pictures. A suite of techniques that join diverse highlights and order procedures have been produced and tried for recognizing femur breaks [38].	The researcher’s next target is to build up a model framework for field tests in the medical clinic.	The favorable aspect of this versatile examinatopm technique is that it requires the extraction of only estimated bone forms. In this way, it can likewise endure a slight variation in shape over various patients; furthermore, it does not require the extremely precise extraction of the bone forms.
S25	The researchers present a principal joined utilization of a few developed prescient models from the field of managed machine learning for hip crack forecast in a populace of DXA scanned people. The archive that groups tree-based models that utilize boosting and bootstrap conglomeration methodologies can enhance oppressive capacities for autonomous subjects and give adequate aligned probabilities with the best dependability for the female companions. They trust that these execution measurements can be further enhanced through the gathering of existing global datasets and longer perception periods [39].	Further enhancements in prescient capacity are likely conceivable with the accumulation of more information and longer perception periods.	Machine learning can enhance hip fracture forecast past calculated relapse utilizing gathering models.
S26	A technique has been developed to precisely gauge the the three-dimensional position and introduction (present) of fake knee embeds in vivo from X-ray fluoroscopy pictures utilizing intelligent 3D PC design. In vitro precision tests demonstrate that the technique is exactly within 0.5 mm of error for interpretations parallel to the picture plane and within 0.35” of introductions about any hub [40].	Researchers have discovered that it is best to fit the femoral part first—at that point, the tibia segment.	The strategy can, on a basic level, be connected to any joint where precise CAD models are accessible.
S27	This investigation has presented a robotized computer-aided diagnostic approach for the identification of OA, which the utilizes a mix of standardizations given presciently, displaying MLR utilization and highlight extraction utilizing ICA. The standardization utilizing MLR permitted us to not only lessen the inter-subject changeability but also expand the partition between CC and OA gatherings. Further investigation uncovered that the proposed framework can provide high-classification execution in recognizing solid and osteoarthritic patients from various knee sides [41].	This CAD framework stays away from the subjectivity and the significance of the administrative skill involved in manual activities and can make predictions based on inconspicuous information.	The classification rates of the proposed CAD are higher than those obtained in past studies.
S28	This research study explores the morphometrical comparison between CT scan databases for miniaturized objects by using the two-division procedure. In all three datasets and both deliberate parameters, measurable differences were found, especially for the demonstration of the expanded parameters of the pictures sectioned by this CV method [42].	The CV calculation is proposed in instances of basic BV/TV esteems because of its protection from the fracture impact.	The primary constraint of the present investigation is the nonappearance of a strong standard against which to compare the diverse outcomes.
S29	This paper attempts to fill the gap with a total and completely programmed system of FEM, displaying and applying it to MDCT pictures. It proposes a space-variant hysteresis picture preparation convention for bone pictures. Cutting-edge strategies for work age are connected as for work quality in the structure. Bone solidness is processed with μ-CT, and MDCT utilizes this system [43].	Real bone solidness is dictated by mechanical testing. Bone anticipated as more solid by FEM was seen to be very reproducible and relate well with that anticpiated so by the mechanical testing. The outcome shows that MDCT can be utilized for FEM recreation.	The system has been effectively connected to the FEM of both μ-CT and MDCT imaging under certain conditions. At present, the technique is explored for MDCT pictures in an Iowa bone improvement study, and the pilot considers information including on subjects from various investigation cohorts with anticipated distinctions in bone digestion.
S30	The goal of the present examination was to utilize fluoroscopy to precisely decide the three-dimensional (3D), in vivo, weight-bearing kinematics of 10 typical and five front cruciate tendon-lacking (ACLD) knees. Persistent explicit bone models were derived from registered tomography (CT) information [44].	The PC-created 3D models of each subject’s femur and tibia are unequivocally enlisted to the 2D computerized fluoroscopic pictures utilizing an improved calculation that naturally changes the posture of the model at different flexion/augmentation edges.	The preference of the present trial demonstrates that it is a permitted investigation under in vivo, weight-bearing conditions for the whole scope of knee flexion.
S31	The primary objective of this study was to assemble a limited component, demonstrate phenomena for trabecular bone from small scale figured tomography (miniaturized scale CT) pictures, and concentrate on the flexible properties of bone. We additionally contemplated the connection between the Young’s modulus and porosity of trabecular bone. Analysts in the past have demonstrated that porosity and clear thickness were the two fundamental factors that influenced the Young’s modulus of trabecular bone [45].	To additionally direct this investigation, tests from different kinds of human bones ought to be planned.	For a limited component examination strategy, various yields can be extricated as required.
S32	In this examination, a few morphological parameters were estimated at the same time for each example as opposed to simply concentrating on a couple of parameters. Thus, the coefficient of assurance expanded the utilization of the different relapses. However, it is still evident that the porosity and obvious thickness unequivocally influence the mechanical properties of trabecular bone [46].	The parameters tended to in this examination are not intently correspondent to one another. Accordingly, including these non-correspondent parameters in the direct various relapses improved the exactness.	Some morphological parameters are identified with one another; the number of parameters could be diminished, generally leaving only obvious thickness

**Table 13:** Deep learning papers.

#	Gaps
S1	By looking at the longitudinal information of the resulting values, a slight weakness of the ligament is seen in the MR cuts, just as in the division results, along these lines, showing the potential clinical utility of the proposed calculation.
S2	In the results of this research, it may very well be observed that the pre-trained models indicate low execution in the errand of bone tumor order determination. The reason is that these modes have experienced an over-fitting issue because of the high number of calculations but fewer measures of information.
S3	The arrangement precision of KL Grades 1 and 2 cannot be observed as solid, noting that the radiographs per user are frequently tested in order to recognize these evaluations, and in this manner, the confusion of the automated identification between these two evaluations cannot be considered astonishing.
S4	There is a need to investigate more feature extraction by using classification for the detection of the knee joint.
S5	The examination of the disorder is done according to both clinical side effects and radiological appraisal. While the authors have considered only the radiological appraisal of knee X-rays, the mischaracterization rate is rather high.
S6	The ordinary picture delivered lower vitality when contrasted with the anomalous picture. The proposed calculation comes up short if the bone part in the X-ray picture is skewed, since the recognizable proof of the focal piece of synovial depression locale strongly relies upon the situation of the bone, and it is required to be immaculate in a vertical way.
S7	The particular element of the bones is the unevenness of power, which can impact the result’s accuracy.
S8	In the OAI information, each of the 88 patients contributes two MR volumes (~1 year, separated), which can freely fall in testing, and preparing the dataset may reveal some inclination in the assessed blunder measurements.
S9	This methodology could not be utilized to use bigger subvolumes or even the full picture for the division of knee bones and ligaments, which could continually be refined by shape information.
S10	The proposed outcome does not indicate a huge enhancement of the precision in surface measurements.
S11	Their study had several limitations. Only the articular ligament on the femur and tibia was assessed, and the division and characterization CNNs were most certainly not streamlined for evaluation in this research.
S12	PSI requires ceaseless enhancement to accomplish better clinical results than have previously been described for routine use.
S13	A few constraints and upgrades that would most likely improve the execution are distinguished. An enhanced yet comparative model may not be helpful in a few clinics, and applications in various medicinal assignments should be looked into.
S14	Additionally, the proposed Method 3 had a blunder rate practically identical to that of Method 1, dependent on the response drive.
S15	This second level continues as a very particular procedure to dismiss troublesome false positives while preserving high sensitivities.
S16	The outrageous work cost of the MRI investigation makes the procedure wasteful and costly.

**Table 14:** Gaps in machine learning papers.

#	Gaps
S17	This present investigation’s impediment includes the two-dimensional examination, restricted age range, and insufficient sizes for a few subgroups.
S18	There is a requirement for increasingly delicate magnetic resource imaging strategies that can recognize inconspcious changes related to pre-radiographic OA and that may conceivably serve as biomarkers for malady initation and movement.
S19	This methodology would expand the multifaceted nature of the calculation.
S20	This study further shows the requirement for the evaluation of multivariate techniques in settings of various attractive fields, temperatures, and heartbeat groupings, as well as in appliction to other ligament models, for example, osteoarthritis and designed tissue.
S21	The interinstitutional order precision, while adequate, was not as high as interinstitutional examination.
S22	The basic piece of the knee bone structure is completely overlooked, which is crucial for finding knee issues.
S23	ASM techniques cannot accomplish satisfactory limitation results, since they ill suit the vast varieties of patellae present among various pictures.
S24	The fraction identification rate of the person classifier is not high.
S25	This methodology is doubtful as it can expel exceptional indicators.
S26	The strategy does not gauge the interpretation opposite to the picture plane and expects that the two knee segments are adjusted toward this path.
S27	The speculation capacities of the proposed strategy could be overestimated and ought to be taken with caution.
S28	The rate of distinction between the used databases in this study was observed to be greater, which prompts typical outcomes and representation of the trabecular bone.
S29	The system is required to be connected in a more extensive territory on imaging.
S30	Picture twisting and non-uniform scaling can happen; these cannot be made up for via watchful alignment.
S31	This investigation has a few limitations. As a matter of first importance, the examples utilized in this investigation are porcine femurs rather than human bone. Even though the macro-structure and micro-structure of porcine bones are fundamentally the same as those of human bones, the creation of trabecular bone is extraordinary.
S32	Some morphological parameters are identified with one another; the number of parameters could be diminished, generally leaving only obvious thickness.

**Table 15:** Deep and machine learning techniques.

Deep Learning Techniques	Machine Learning Techniques
CNN SSEW Kellgren–Lawrence classification The active contour segmentation technique SSLBP SSMs Finite Element FE Sobel operator Canny edge detection Laplacian of Gaussian operator K-Nearest Neighbor Algorithm (KNN)	General Linear Model WND-CHARM SVM Multivariate Support Vector Machine Sobel Edge Detector Operator Bayesian Classifier Bootstrap Aggregation Computer-aided Design Finite Element Method Multivariate Linear Regression (MLR)

**Table 16:** Accuracy.

	X-ray	MRI	CT Scan
Deep Learning	87.7%	91.8%	86.24%
Machine Learning	75.9%	87.8%	77.6%

**Table 17:** Accuracy of deep learning papers.

Studies	Datasets	X-Ray	MRI	CT Scan
S1	3000 subjects from the Osteoarthritis Initiative dataset	66.71%	No	No
S2	ImageNet dataset	97.64%	No	No
S3	350 images of X-rays	84.72%	No	No
S4	Bilateral PA fixed flexion knee X-ray images	94.2%	No	No
S5	200 datasets of knee X-ray images	87.92%	No	No
S6	X-ray images of OA patients	94.9%	No	No
S7	Online MRI dataset	No	96.1%	No
S8	MICCAI datasets	No	87.7%	No
S9	3D MRI datasets SKI10 Dataset OAI Imorphics Dataset OAI ZIB	No	88.21%	No
S10	MRI datasets of knee	No	95.9%	No
S11	MRI datasets of 175 patients	No	91%	No
S12	CT scan and MRI data collected from multiple types of research	No	No	95%
S13	Dataset of 21 abdominal CT scan images	No	No	93%
S14	CT scan data of multiple patients	No	No	83.23%
S15	CT images of 59 patients	No	No	80%
S16	3D image data of knee joints	No	No	80%
Total Accuracy		87.7%	91.8%	86.24%

**Table 18:** Accuracy of machine learning papers.

Studies	Datasets	X-Ray	MRI	CT Scan
S17	Tested reports of Gaucher patients	No	70%	No
S18	35 MRI images	No	86%	No
S19	Dataset of 18 images	No	91%	No
S20	(1) 36 BNC samples, (2) 40 control samples	No	97%	No
S21	Knee MRI reports	No	95%	No
S22	X-ray images of patients	50%	No	No
S23	(1) 21 images for training patella, (2) 48 images as the validation set	80%	No	No
S24	432 images of the femur	97.2%	No	No
S25	75,000 patients’ data	95%	No	No
S26	24 X-ray images	50%	No	No
S27	1024 X-ray images	82.98%	No	No
S28	Micro CT trabecular datasets	No	No	65%
S29	Multidetector Computed Tomography (MDCT) dataset	No	No	98%
S30	10 knees-experienced patterns	No	No	90%
S31	Seven samples of pig femur bone	No	No	65%
S32	CT scan images of patients	No	No	70%
Total Accuracy		75.9%	87.8%	77.6%

## Appendix A

Primary Studies of Comparative Systematic Literature Review:

**Table A1:** Reviewed papers and references.

#	Citation	#	Citation
S1	[4]	S17	[32]
S2	[19]	S18	[33]
S3	[20]	S19	[7]
S4	[21]	S20	[34]
S5	[22]	S21	[35]
S6	[23]	S22	[36]
S7	[24]	S23	[37]
S8	[25]	S24	[38]
S9	[26]	S25	[39]
S10	[5]	S26	[40]
S11	[27]	S27	[41]
S12	[28]	S28	[42]
S13	[6]	S29	[43]
S14	[29]	S30	[44]
S15	[30]	S31	[45]
S16	[31]	S32	[46]
